# Limitations in the isolation and stimulation of splenic mononuclear cells in a dasyurid marsupial, *Phascogale calura*

**DOI:** 10.1186/s13104-018-3824-5

**Published:** 2018-10-10

**Authors:** C. Letendre, L. J. Young, J. M. Old

**Affiliations:** School of Science and Health, Western Sydney University, Locked Bag 1797, Penrith, NSW 2751 Australia

**Keywords:** Marsupials, Dasyurid, Spleen, PBMC, Mononuclear cells, Cell culture, Mitogen, Phytohemaglutinin

## Abstract

**Objective:**

Marsupials suffer from an increasing number of stressors in this changing world. Functional studies are thus needed to broaden our understanding of the marsupial immune system. The red-tailed phascogale (*Phascogale calura*) is a small Australian marsupial previously used in descriptive immunological studies. Here, we aimed to develop functional assays by isolating and stimulating blood and spleen mononuclear cells in vitro.

**Results:**

While peripheral blood mononuclear cell (PBMC) were relatively easy to isolate, only 10^5^ mononuclear cells (> 90% purity and > 75% viability) could be recovered from the spleen, independently of the sex and age of the animal or the centrifugation time and speed tested. The pores of the mesh sieve used for tissue homogenization might have been too big to yield a single cell suspension. Nevertheless, in spite of the overall low number of cells recovered, PBMC and splenic mononuclear cells were successfully activated in preliminary trials with phytohemaglutinin. This activation state was evidenced by a change in shape and the presence of small cell aggregations in the mitogen-stimulated cultures. A non-radioactive colorimetric assay was also performed to confirm cell proliferation in these wells. This work highlights the importance of developing and reporting detailed methodological protocols in non-traditional research species.

## Introduction

Marsupials suffer from environmental and anthropogenic stressors that pose pressure on their immune system [1, 2]. Functional immunological studies are thus needed to develop relevant conservation strategies. The marsupial immune system has long been regarded as ‘primitive’, with reports of weak or delayed adaptive responses compared to eutherians [3–13]. However, these differences might simply be due to the use of suboptimal assay conditions. There is now a growing body of evidence suggesting that the marsupial immune system is just as intricate and complex as that of eutherians [14].

Lymphocytes proliferate and become activated in response to antigens encountered in secondary lymphoid organs (e.g. spleen). Activated lymphocytes (CD4^+^ T cells in particular) produce cytokines and these immune-regulatory molecules determine the type of response generated [14]. Although mitogen stimulation induces non-specific activation, these assays have been used extensively in marsupials to investigate lymphocyte ‘fitness’ and their proliferative capacities (Table 1). Mitogen stimulation assays can be conducted on total splenocytes. However, characterization of the lymphocyte cytokine profile in response to certain stimuli is facilitated by the use of isolated mononuclear cells. In eutherians, splenic mononuclear cells are commonly isolated by density gradient separation after mechanical disruption and passage on a mesh sieve [15]. While some work has been done with marsupial peripheral blood mononuclear cells (PBMC), mononuclear cells have only been isolated occasionally from lymphoid tissues (Table 1). This is due in part to the ethical limitations associated with collecting tissues from threatened/endangered animals, but most importantly to the low variety of tools and protocols available for these species [14].

**Table 1:** Mitogen stimulation in marsupials

Species	Tissue or cell type	Cell stimulant	Application	Refs.
*Dendrolagus matschiei*	Whole blood	PHA, ConA, PWM	[3H]-thymidine	[8]
*Didelphis virginiana*	Blood leukocytes	PHA, ConA, PWM	[3H]-thymidine	[13]
*Macropus eugenii*	PBMC, spleen MC and LNMC	PHA	[3H]-thymidine and MTT	[25]
	LNMC	PHA, ConA	RNA extraction	[26, 27]
	PBMC, LNMC	Zimosan, PHA, LPS, PWM	RNA extraction	[28–31]
*Monodelphis domestica*	PBMC	ConA, PHA, PWM	[3H]-thymidine	[32]
	Whole blood, spleen, LN, thymus, peritoneal M$\upphi$, skin	PHA, ConA, PWM, LPS	[3H]-thymidine	[33, 34]
	Whole blood	ConA	[3H]-thymidine	[6]
*Onychogalea fraenata*	Spleen MC	PHA	RNA extraction	[31]
*Perameles gunnii*	PBMC	ConA, PHA, PWM, LPS	[3H]-thymidine	[35]
*Phascolarctos cinereus*	Whole blood, PBMC	PHA and ConA, PWM, LL, jacalin, PA and LPS	[3H]-thymidine	[9]
	PBMC	Ionomycin, PMA	Flow cytometry	[36]
	PBMC	PMA and ionomycin, PMA and PHA, ConA	RNA extraction	[37, 38]
	PBMC	PMA and ionomycin, *C. pecorum*	RNA extraction	[39–41]
*Sarcophilus harrisii*	PBMC	ConA, PHA, PWM, LPS	[3H]-thymidine	[35, 42]
	PBMC	Poly(I:C), LPS, flagellin, imiquimod, CpG, profiling	RNA extraction	[43]
*Setonix brachyurus*	PBMC	PHA	[3H]-thymidine	[44]
	Blood leukocytes, spleen, thymus, LN	PHA, ConA, PWM	[3H]-thymidine	[45–47]
*Trichosurus caninus*	PBMC	Ionomycin, PMA	Flow cytometry	[36]
*Trichosurus vulpecula*	Spleen	PHA	[3H]-thymidine	[3, 4]
	PBMC	PHA	[3H]-thymidine	[48]
	PBMC, spleen, AM	*M. bovis*, LPS, LAM	[3H]-thymidine, RNA extraction	[49–51]
	PBMC	Ionomycin, PMA	Flow cytometry	[36]
	PBMC, spleen MC, LNMC	ConA	[3H]-thymidine, RNA extraction	[52]

*AM* alveolar macrophages, *ConA* concanavalin A, *CpG* CpG oligodeoxynucleotide, *LL* lentil lectin, *LN* lymph nodes, *LNMC* lymph node mononuclear cells, *LPS* lipopolysaccharides, Mϕ macrophages, *MC* mononuclear cells, *PA* protein A, *PBMC* peripheral blood mononuclear cells, *PHA* phytohemaglutinin, *PMA* phorbol myristate acetate, *PWM* pokeweed mitogen

The red-tailed phascogale (*Phascogale calura*) is an Australian daysurid. This small carnivorous marsupial breeds in captivity, which makes it an ideal research model [16–22]. The species is best known for its particular life history, where males die shortly after the breeding season [23]. In captivity, males survive over one year of age, but become infertile and develop signs of accelerated aging and immunosenescence ([24], unpublished results). Given the important role of lymphocytes in the coordination of adaptive immunity, we aimed to isolate and stimulate these cells in vitro. Here, we show the limitations encountered while standardizing a protocol for isolation and stimulation of phascogale splenic mononuclear cells. The end goal of this research is to investigate the type and magnitude of cytokines produced across different age-sex groups. Demonstration of an impaired lymphocyte function in captive adult males would pave the way for future studies of immunosenescence in marsupials.

## Main text

### Methods

#### Animals

Red-tailed phascogales were sourced from a captive breeding colony housed in the Western Sydney University (WSU) School of Science and Health Native Mammal Teaching and Research Facility, Richmond, NSW, Australia. Animal care was as per the Australian Code for the Care and Use of Animals for Scientific Purposes and the New South Wales Animal Research Act and its Regulations [24]. Protocols were approved by the WSU Animal Care and Ethics Committee (A11197). All samples were collected opportunistically from clinically healthy animals euthanized during routine population management procedures.

#### Cell isolation

Blood and spleen were collected aseptically, immediately after euthanasia with CO_2_. Samples were carried to the laboratory in an insulated cooler and were processed within 30 min of collection.

*Blood* Blood was collected via heart puncture (about 1 ml/animal) and placed into sodium EDTA-coated tubes. PBMC were isolated on a density gradient [25]. Blood was diluted with phenol red-free, endotoxin-free Hanks balanced salt solution with calcium and magnesium (HBSS+) (Sigma, St. Louis, MO, USA). The final 4 ml suspension was layered onto 2 ml Ficoll-Paque (GE LifeSciences, Pittsburgh, PA, USA) (1.077 g/ml). PBMC were collected at the interface after centrifugation (400*g*, 25 min). To confirm isolation of mononuclear cells, smears were made for each layer of the gradient using 2-µl drops and Diff-Quik staining (Bacto Laboratories, Australia). PBMC were washed with HBSS+. A tris-buffered ammonium chloride solution (0.16 mol/l ammonium chloride, 0.01 mol/l tris(hydroxymethyl)aminomethane (Tris), pH 7.3) was used for red blood cell (RBC) lysis. After additional washing steps, PBMC were resuspended in 100–200 µl culture medium (see below). Cell count and viability were evaluated manually by Trypan blue exclusion. Mononuclear cell purity was assessed on Diff-Quik stained smears.

*Spleen* The spleen was transported into HBSS+ containing 200 U/ml penicillin, 200 µg/ml streptomycin and 100 µg/ml gentamicin. It was placed on a sterile 60-mesh stainless-steel sieve (pore size 250 µm) onto a collector tube, perfused with culture medium, teased apart using micro-dissecting scissors, and gently pushed through the sieve using a syringe plunger. The final volume of the splenic suspension was 4 ml. Total WBC counts were determined on an aliquot using Türk’s solution (0.01% crystal violet, 1% glacial acetic acid), which lyses RBC and stains leukocytes to assist counting [53]. The splenic suspension was layered onto 2 ml Ficoll-Paque and mononuclear cells were recovered at the interface following centrifugation (see conditions tested in Table 2). Finally, cells were washed and counted as described for PBMC. The recovery rate (%) corresponds to (number of mononuclear cells isolated)/(total number of WBC) × 100.

**Table 2:** Conditions tested for isolation of splenic mononuclear cells

Date	Sex (number of individuals pooled)	Age (months)	Conditions tested			Spleen cells		
			Speed (*g*)	Time (min)	RBCL	WBC count	Recovery (%)	Viability (%)
18 April 2017	F (1)	9	400	25	No	3.5 × 10^7^	0.5	86
2 May 2017	M (1)	10	400	20	Yes	8.9 × 10^7^	0.2	97
22 May 2017	M (1)	10	400	25	Yes	1.7 × 10^7^	0.6	76
	M (1)	10	400	25	Yes	7.1 × 10^7^	1.2	82
29 June 2017	F (1)	11	400	25	Yes	6.5 × 10^6^	0.5	88
6 July 2017	M (2)	12	400	25	Yes	3.2 × 10^7^	0.1	72
18 July 2017	F (3)	12	400	25	Yes	8.8 × 10^7^	0.1	88
25 July 2017	F (2)	12	400	20	Yes	1.9 × 10^7^	0.0	68
	M (2)	12	400	20	Yes	5.0 × 10^7^	0.1	88
1 Aug 2017	M (2)	13	350	20	Yes	1.4 × 10^7^	0.3	88
	M (2)	13	400	15	Yes	1.5 × 10^7^	0.2	75
23 Aug 2017	F (2)	13	400	15	No	2.3 × 10^7^	1.0	87
8 Sept 2017	F (2)	14	400	15	No	3.1 × 10^7^	0.1	78
4 Oct 2017	F (1)	4	400	15	No	2.1 × 10^7^	0.7	85

#### Cell culture and stimulation

Cells were diluted to the desired concentration in Iscove’s Modified Dulbecco’s Medium (IMDM) with GlutaMAX supplement, 100 U/ml penicillin, 100 μg/ml streptomycin, 50 μg/ml gentamicin and 5–10% fetal calf serum (FCS; LifeTechnologies, Carlsbad, CA, USA). Cells were plated in adherent, flat bottom, 96-well plates. Plates were incubated at 35 or 37 °C, 5% CO_2_ for 24–72 h. Cells were visualized using an Olympus CKX41 microscope. Culture conditions yielding the best viability were selected for the mitogen stimulation assay. Cells were stimulated with phytohemaglutinin from *Phaseolus vulgaris* (PHA-M; 10, 25 or 50 µg/ml; Sigma), based on concentrations reported for other species [3, 9, 25, 34, 35].

#### Quick Cell Proliferation Assay

The Quick Cell Proliferation Assay kit II (Abcam, Cambridge, UK) was used to quantify cell proliferation and viability of mitogen-stimulated cells, as per the manufacturer’s instructions. The plate was read at 450 nm using a multi-well spectrophotometer (Biorad, Hercules, CA, USA) after 4 h incubation.

#### Statistics

A one-way ANOVA was used to analyze the effect of PHA concentration on PBMC proliferation, and a two-way ANOVA for the effect of cell density and PHA treatment. *p*-values were Bonferroni-adjusted. The effect of PHA stimulation on splenocyte proliferation was analyzed using an independent sample T-test. Analyses were performed with IBM SPSS Statistics software (v.24). *p* < 0.05 was considered as statistically significant. Data are mean ± SEM, unless otherwise stated.

### Results and discussion

#### Isolation of splenic mononuclear cells

The protocol used here for isolation of splenic mononuclear cells is based on previous work in the tammar wallaby (*Macropus eugenii*) [25, 53], but was found to be suboptimal in the phascogale. In this study, only about 10^5^ mononuclear spleen cells (> 90% purity and > 75% viability) were routinely recovered, independently of the sex and age of the animal or the technical conditions tested (Table 2). The phascogale spleen is obviously much smaller than that of a wallaby, but the number of cells recovered was still low compared to a murine spleen of similar size ([15], personal observation). Marx et al. similarly reported a low cell density for the opossum (*Didelphis virginiana*) spleen compared to mice [12]. To determine whether the low cell numbers obtained in this study were due to an inherent low cellular density or rather to a suboptimal isolation technique, we evaluated the total number of white blood cells (WBC) in the splenic suspension prior to the density gradient separation. We found that the phascogale spleen contained on average 3.7 ± 0.7 × 10^7^ WBC, which means that only 0.4 ± 0.1% of these cells were effectively recovered (Table 2). In comparison, > 10% PBMC were routinely recovered under similar conditions: centrifugation of whole blood yielded a clear white band at the interface of the density gradient, which facilitated the recovery of mononuclear cells. Meanwhile, only a diffuse smear in the lower phase of the gradient was obtained with the splenic suspension. Spleen cells are highly heterogeneous: they could vary in their buoyancy and separate non-uniformly on the density gradient. Interestingly though, the number of splenic mononuclear cells recovered remained low with shorter centrifugation times (Table 2; 1 August 2017). This persistent non-uniform cell distribution could be explained by an inadequate homogenization and separation of the splenic tissue on the sieve. A sieve with 200-μm pores is typically recommended in mice for preparation of lymphoid cell suspensions [15]. However, we used a 250-µm sieve as described in the wallaby [25]. The sieve pores were therefore possibly too big to yield a single cell suspension. In fact, cell clumps were often visualized in smears from the lower phase of the gradient. In contrast, no such clumps were visualized during PBMC isolation. While the pore size of the mesh sieve appears critical for preparation of single cell suspensions, this information is rarely mentioned in the marsupial literature and should be included in future publications. At this stage, it also remains unclear whether the mononuclear cells of the phascogale and wallaby differ in their buoyancy, but this type of interspecies variation is well documented in eutherians [54]. Finally, since most RBC were removed by centrifugation, RBC lysis was eventually eliminated from the protocol to avoid any further cell loss and unnecessary damage (Table 2).

#### Culture of splenic and peripheral blood mononuclear cells

Given the low number of cells routinely recovered, the conditions tested for splenic mononuclear cells were based on those determined in pretrials with PBMC. Early standardization work was carried out as a series of small experiments testing only one variable at a time. Temperature was found to affect cell viability in vitro. It was drastically impaired when cells were incubated at 37 °C (15% after 24 h) compared to 35 °C (92% after 48 h, and 76% after 72 h). Lymphoid cells from another dasyurid, the Tasmanian devil (*Sarcophilus harrisii*), have been cultured similarly at 35 °C [43]. Marsupials typically have a lower body temperature (by 1.0–3.0 °C) and a lower basal metabolic rate (by 30%) than eutherians [55]. However, marsupial cells are still generally cultured at 37 °C [4, 9, 25, 32, 36, 46]. The reasons for these interspecies variations remain unclear but may depend on the culture medium/buffer. Moreover, cell viability was density-dependent: good viability was obtained after 48–72 h with relatively high cell densities (0.5–1 × 10^6^ cells/ml in 150–200 µl), as described in the wallaby [52]. Finally, different concentrations of FCS were tested [13], but viability remained unchanged at concentrations above 5% FCS.

#### Mitogen stimulation and assessment of proliferation

**Fig. 1:**
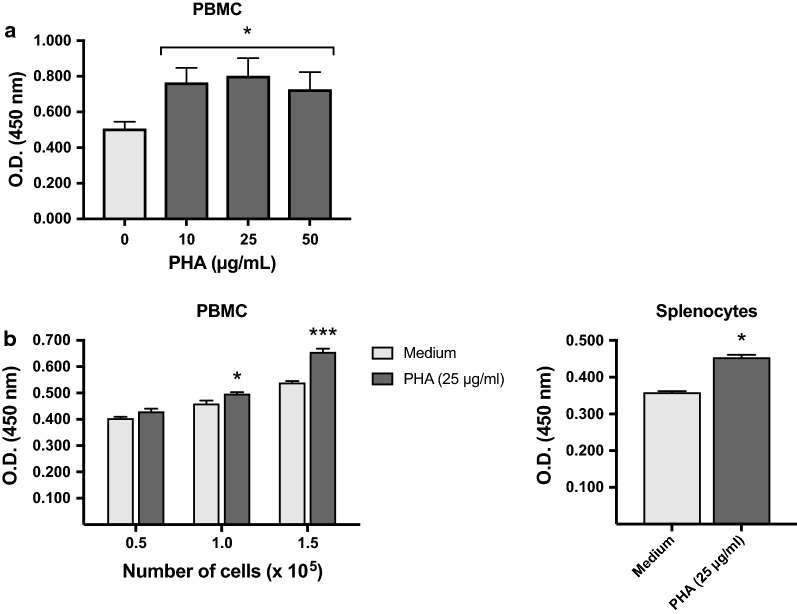
Cell proliferation in response to stimulation with phytohemaglutinin (PHA). 2 × 10^5^ PBMC were stimulated with different concentrations of PHA (final volume = 150 µl). After 48 h, reagent from the Quick Cell Proliferation Assay kit was added to each well and absorbance of the dye solution was measured at 450 nm after an additional 4 h of incubation. The graph shows the dose–response curve obtained for two individuals (**a**). 0.5–1.5 × 10^5^ PBMC or 1 × 10^5^ splenic mononuclear cells were stimulated with PHA (25 µg/ml; final volume = 150 µl) and proliferation was assessed after 48 h using the Quick Cell Proliferation Assay kit, as described in **a**. Work was performed in duplicates and data are expressed as mean ± SEM (**b**). Asterisks indicate a statistically significant difference (*p < 0.05 and ***p < 0.0005)

PBMC and splenic mononuclear cells progressively adopted an activated, Y-shaped morphology within 4–6 h of PHA stimulation [25]. Numerous small cell aggregations were also present after 24 h in mitogen-stimulated cultures. As accurate cell counts cannot be determined manually by Trypan blue exclusion when cells are aggregated, proliferation assays are routinely used (Table 1). The Quick Cell Proliferation Assay is a non-radioactive colorimetric approach where the amount of dye generated is proportional to the number of living cells [56]. Increased proliferation was observed both in PHA-stimulated PBMC and splenic mononuclear cell cultures (*p* = 0.020 and *p* = 0.023, respectively) (Fig. 1a, b). However, no dose–response was observed with increasing concentrations of PHA (*p* = 0.190). The strongest effect of mitogen stimulation was observed at high cell densities (*p* < 0.0005) (Fig. 1b). Similar results were obtained with splenic mononuclear cells, although only selected conditions could be tested with this cell type.

## Limitations and conclusions

This work highlights the importance of developing rigorous methodological protocols in non-traditional research species as suboptimal assay conditions are likely to yield biased comparisons with other species. Technical details for isolation of lymphoid cells (sieve pore size, centrifugation time and speed) should be consistently reported in future publications to ensure repeatability across studies. Here, we offer preliminary results showing successful lymphocyte activation and proliferation in response to mitogen stimulation in the phascogale. Future studies investigating the impact of age and sex on lymphocyte function in this species will depend on the capacity to further increase the recovery of mononuclear cells. FACS sorting based on cell size and granularity might be a promising alternative.

## Abbreviations

AM: alveolar macrophages
ConA: concanavalin A
CpG: CpG oligodeoxynucleotide
LL: lentil lectin
LN: lymph nodes
LNMC: lymph node mononuclear cells
LPS: lipopolysaccharides
M$$\upphi$$: macrophages
MC: mononuclear cells
PA: protein A
PBMC: peripheral blood mononuclear cells
PHA: phytohemaglutinin
PMA: phorbol myristate acetate
PWM: pokeweed mitogen
RBC: red blood cells
WBC: white blood cells
